# The Role of Body Image on Psychosocial Outcomes in People With Diabetes and People With an Amputation

**DOI:** 10.3389/fpsyg.2020.614369

**Published:** 2021-01-11

**Authors:** Sarah McDonald, Louise Sharpe, Carolyn MacCann, Alex Blaszczynski

**Affiliations:** ^1^Graduate School of Health, University of Technology Sydney, Sydney, NSW, Australia; ^2^Clinical Psychology Unit, University of Sydney, Sydney, NSW, Australia; ^3^School of Psychology, University of Sydney, Sydney, NSW, Australia

**Keywords:** amputation, diabetes, body image, quality of life, anxiety, depression, psychosocial

## Abstract

**Introduction:**

Research indicates that body image disturbance is associated with poorer psychosocial outcomes for individuals with physical health conditions, with poorest body image reported for individuals with visible bodily changes. Using White’s (2000) theoretical model of body image the present paper aimed to examine the nature of these relationships in two distinct groups: individuals with an amputation and individuals with diabetes. It was hypothesized that body image disturbance would be associated with psychosocial outcomes and would mediate the relationships between self-ideal discrepancy and personal investment in psychosocial outcomes.

**Methods:**

Individuals with diabetes (*N* = 212) and individuals with an amputation (*N* = 227) provided details regarding their medical condition, and completed measures assessing body image, investment, self-ideal discrepancy, depression, anxiety, and quality of life. Structural equation and invariance modeling were used to test the model paths and the invariance of the model.

**Results:**

As hypothesized, body image disturbance was found to mediate the relationships between personal investment and psychosocial outcome, and between self-ideal discrepancy and psychosocial outcome. The predicted paths were invariant across groups, although the model accounted for more variance in people with an amputation than people with diabetes.

**Conclusion:**

Body image disturbance, personal investment, and self-ideal discrepancy are important factors contributing to psychosocial outcome for individuals with diabetes and individuals with an amputation. These findings not only confirm the validity of the model in these two groups, but they emphasize the importance of targeting body image in future psychological interventions for individuals with a health condition.

## Introduction

Body image is a relatively neglected topic in patients with health problems, despite the fact that most health problems have the potential to change a person’s body, whether as a direct result of the illness or its treatment. Where body image has been studied in health contexts, typically investigators have: (a) compared individuals with an illness to a healthy control group and found that body image is compromised across a range of medical conditions (e.g., Cornwell and Schmitt, 1990; Schiavi et al., 1995; Sarwer et al., 1999; Yuen and Hanson, 2002; Lazaridou-Terzoudi et al., 2003; Weinstein et al., 2003; Huang et al., 2006; Noyan et al., 2006; Abbott et al., 2007; Kaymak et al., 2007; Moin et al., 2009; Guenther et al., 2010; Versnel et al., 2010; Blashill and Wal, 2011; Steinmann et al., 2011; Versnel et al., 2011) and/or (b) found a relationship between body image disturbance and psychosocial outcomes (e.g., Rybarczyk et al., 1995; Breakey, 1997; Carver et al., 1998; Eiser et al., 2001; Marcusson et al., 2002; Perez et al., 2002; Taleporos and McCabe, 2002; Benrud-Larson et al., 2003; Petronis et al., 2003; Limb, 2004; Tebble et al., 2004; Coffey et al., 2009; Fingeret et al., 2011; Sharpe et al., 2011; Bullen et al., 2012; Teo et al., 2018; Fang et al., 2019; Pieta et al., 2020). However, very little of this research has been driven by theoretical models that have asked questions about the nature of body image disturbance and the way in which body image factors likely contribute to poorer psychosocial outcomes (Feragen and Stock, 2018).

Although there are many models of body image for the general population, White’s (2000) Heuristic Cognitive Behavioral Model of body image for oncology patients remains the only theoretical model of body image developed specifically for patients with a chronic illness (Pruzinsky, 2004). White suggests that any bodily change will be processed in the context of an individual’s pervasive beliefs about themselves (i.e., self-schema) that are triggered by a real or perceived change in their appearance. According to White’s model, these schemas will determine the degree of importance individuals place on their appearance. People who have negative self-schema and are invested in their bodily appearance become invested in the changed body part, which contributes to the development of the self-ideal discrepancy. White (2000) proposes that negative body image schemas, investment in appearance and a self-ideal discrepancy lead to the activation of negative appearance-related assumptions, negative automatic thoughts and/or images, maladaptive behaviors, and ultimately emotional distress. Both emotional distress and unhelpful behaviors (such as avoidance) in turn maintain the negative schemas and investment in body ideals.

White’s (2000) model built upon the connections described by cognitive behavioral accounts of body image (Cash and Szymanski, 1995; Cash, 2002, 2011), with specific attention to the impact of cancer. In doing so, White’s (2000) model was the first to provide a hypothesis-generating conceptual framework for understanding the possible etiology of body image disturbance in oncology. Numerous studies in oncology provide support for one or more of the central tenets of White’s model in a range of cancers, including breast cancer (Petronis et al., 2003; Figueiredo et al., 2004; Metcalfe et al., 2005; Browall et al., 2008; Dahl et al., 2010; Moreira and Canavarro, 2010; Collins et al., 2011), colorectal cancer (Cotrim and Pereira, 2008; da Silva et al., 2008; Sharpe et al., 2011), prostate cancer (Perez et al., 2002), head/neck cancer (Fingeret et al., 2011), oral cavity cancer (Fingeret et al., 2010), osteo- or ewings-sarcoma (Eiser et al., 2001), gynecological cancer (Teo et al., 2018), and melanoma (Lichtenthal et al., 2005). All of these studies provide support for the strong association between body image disturbance and poor psychosocial outcomes in cancer. Moreover a number of these studies employed prospective designs and found that body image was a significant predictor of future psychosocial outcome (Shimozuma et al., 1999; Browall et al., 2008; Moreira and Canavarro, 2010; Sharpe et al., 2011; Bullen et al., 2012; Taylor-Ford et al., 2012). However, most of the literature, to date, has tested only some of the simple relationships between variables described in White’s model and only in oncology groups. The full model has not been examined using path analysis or structural equation modeling, nor has the model been tested extensively outside of the cancer literature, despite suggestions that it could be equally relevant to other illnesses (Pruzinsky, 2004). Therefore, the above mentioned are the overarching aims of the current study.

Research across a range of illnesses has supported the contention that health related changes in appearance have a negative impact on body image; including pectus excavatum (Steinmann et al., 2011), HIV (Huang et al., 2006; Blashill and Wal, 2011), facial cleft (Marcusson et al., 2002; Versnel et al., 2010, 2011), craniofacial abnormality (Sarwer et al., 1999), ankylosing spondylitis (Guenther et al., 2010), arthritis (Pieta et al., 2020), physical disability (Moin et al., 2009), dermatological conditions (Kaymak et al., 2007), breast cancer (Noyan et al., 2006), cystic fibrosis (Abbott et al., 2007), burns (Thombs et al., 2008), and scoliosis (Weinstein et al., 2003). Perhaps unsurprisingly, illnesses that result in a highly visible bodily changes to the chest and face appear to have a greater negative impact on body image (Marcusson et al., 2002; Versnel et al., 2010; Steinmann et al., 2011). Therefore, although the perception of one’s body and any resulting change is no doubt important, it may also be important to highlight the importance of actual bodily change on body image experience. Interestingly, White’s model indicates that personal investment is a direct predictor of psychosocial outcome. While relationships between personal investment and psychosocial outcomes have been found in studies that have assessed these constructs, the relationship appears to be an indirect one (Lawrence et al., 2004, 2006; Thombs et al., 2008; Moreira and Canavarro, 2010; Partridge and Robertson, 2011). That is, personal investment appears to predict subjective body image disturbance, which in turn predicts psychosocial outcomes. Hence, this suggests that it would be appropriate to include an assessment of subjective body image to better understand these relationships.

On the basis of available research, we aim to examine White’s model of body image disturbance with two distinct but related health groups. We planned to examine two distinct pathways in which objective bodily changes (due to illness, disability, and/or associated treatment) influence subjective body image experience. That is, objective changes can directly influence body image, however, they can also indirectly influence body image experience through increasing personal investment and self-ideal discrepancy. Secondly, based on the broader health literature, we also planned to examine the role of subjective body image experiences. Specifically, how subjective body image is directly influenced by the bodily changes, and how it is also indirectly influenced by the level of investment and self-ideal discrepancy. We hypothesized that subjective body image will be directly associated with psychosocial outcomes and will mediate the relationship between personal investment and self-ideal discrepancy and psychosocial outcomes.

The aim of the present study was therefore to test the validity of White’s (2000) model in two related health groups with rising prevalence rates. To test this model, we selected two health groups, which differ with respect to the degree of visible objective bodily changes. These groups were individuals with diabetes and individuals with an amputation.

Diabetes Mellitus (DM) is a common metabolic disorder with not only increasing prevalence rates observed over the past decade, but according to the World Health Organization the estimated global prevalence rate of diabetes will be 4.4% by the year 2030. In 2017, there were 5 million deaths due to diabetes and the cost of diabetes that year was in excess of $US50 million (Cho et al., 2018). Empirical research demonstrates that body image is strongly associated with depression (Carroll et al., 1999; Ali et al., 2006), poorer treatment adherence (Carroll et al., 1999; Ritholz et al., 2010; Chao et al., 2012), and DM-related complications (Shaban, 2010; Williams et al., 2011, 2013; McDonald et al., 2014). While the presence of complications can cause clear objective and visible bodily changes, such as amputation, many people with diabetes do not have observable change in appearance as a result of their illness. Nevertheless, these results indicate that body image in diabetes has a major impact not only on psychological factors, but also on illness variables. Given that body image may have a role in the illness trajectory for individuals with diabetes, a clear theoretical framework from which to develop interventions to support those individuals where body image is compromised is important.

An amputation is marked by a clear visible and objective change to the body, which can be caused by a disease process (i.e., complication associated with diabetes), but also by trauma. Individuals with an amputation commonly experience symptoms of depression and anxiety within the first 2 years following amputation, however, when this persists it has been associated with poorer physical rehabilitation (Horgan and MacLachlan, 2004). Notably, research indicates that presence of negative body image is significantly associated with prolonged experiences of depression, anxiety, lower QOL, and activity restriction (Breakey, 1997; Coffey et al., 2009; Zidarov et al., 2009).

The use of two different illness groups with differing levels of objective change in bodily appearance allows us to investigate the relevance of body image disturbance in different presentations. Furthermore, it is unknown whether the White’s heuristic model applies solely to populations with observable physical changes to their body as a result of illness (e.g., women with breast cancer following surgery) or whether it also applies in illnesses where there is no identifiable change in appearance due to the illness.

The objective of the present study was to examine the validity of White’s model for individuals with diabetes and individuals with an amputation. It was predicted that structural pathways proposed in White’s model would provide a good fit to the data for both groups. Specifically, it is proposed that body image disturbance, personal investment, and self-ideal discrepancy will all directly predict psychosocial outcome. It was further hypothesized that the total variance accounted for will be greater in the amputation group than the diabetes group given the objective visible bodily changes experienced by this group. It was also predicted that body image disturbance in both groups will mediate the relationships between (a) personal investment and psychosocial outcome, and (b) self-ideal discrepancy and psychosocial outcome. Finally, it is predicted that the structural pathways under examination will be invariant across groups.

## Materials and Methods

### Participants

#### Individuals With Diabetes

Study participants were sent a mailed invitation by a diabetes member’s organization to contact the research team if they were interested in participating in the study. Members invited were over the age of 18 years and had a diagnosis of type 1 or type 2 diabetes. This invitation was sent out to a random selection of 1200 members from their database, from which 389 responded with an expression of interest. All 389 individuals were sent the study pack via their preferred mode of correspondence (mail, email, online, or fax), and 241 (62%) returned the questionnaires. Twenty-nine cases were excluded due to missing data. Therefore, only completed questionnaires (*N* = 212; 88%) were included for the path analysis.

#### Individuals With an Amputation

Individuals who were patients of a local hospital, a prosthetics clinic, and members’ organizations were invited to participate in the study. Inclusion criteria were over the age of 18 years, proficiency in English, and history of an amputation. Total recruitment across sites was 227 participants. The present sample was a convenience sample of individuals who had an amputation. Forty-three (22% of those approached) individuals were recruited from a hospital setting. The main reason for refusal voiced was that the individual felt too medically compromised (multiple medical comorbidities or surgical complications) to complete the study. To increase the range in stage of rehabilitation of the sample, patients of a local prosthetics clinic affiliated with the hospital were also invited to take part, of these 161 individuals completed all measures (82% response rate). Finally, 22 individuals responded to advertisements from members’ organizations, and all (100% response rate) completed the measures. Study participants from prosthetics clinics were sent a mailed invitation from the research team, which asked them to contact the research team directly if they were interested in participating in the study. Study participants from member’s organizations received the invitation via newsletter, which asked them to contact the research team directly if they were interested in participating in the study.

### Ethics

The University’s and Area Health Service’s institutional human research ethics committees both approved the study. The measures are described below.

### Measures

#### Demographics, Medical, and Lifestyle Questionnaire

A questionnaire was constructed to gather information about the individual’s demographic, medical, and lifestyle information. For the DM group this was modeled on the questions asked in the AusDiab study (Dunstan et al., 2002). It included questions about the diagnosis, duration of illness, diabetes related complications (including amputation), treatment, and adherence. For individuals with an amputation this included, time since amputation, cause of amputation, and site of amputation, prosthesis, and pain.

#### Hospital Anxiety and Depression Scale (HADS)

The HADS (Zigmond and Snaith, 1983) is a measure of depression and anxiety symptomatology specifically designed for medical in-patient populations as it relies considerably less on somatic symptoms of depression. The measure has 14 items in total, 7 items measure depression (Cronbach’s alpha = 0.81 for the diabetes group and 0.78 for the amputation group) and the remaining 7 items measure anxiety (Cronbach’s alpha = 0.87 for the diabetes group and 0.82 for the amputation group). For both scales higher scores indicate more depression and anxiety. Scores greater than 8 (out of a possible 21) are said to indicate clinically relevant symptoms of depression or anxiety. Approximately 21% of individuals with an amputation and 18% of individuals with diabetes endorsed clinically significant levels of depressive symptoms (Zigmond and Snaith, 1983). Approximately 25% of individuals with an amputation and 27% of individuals with diabetes reported anxiety symptoms above clinical cut offs (Zigmond and Snaith, 1983).

#### World Health Organization Quality of Life-Brief (WHOQOL-BREF)

The WHOQOL-BREF (Murphy et al., 2000) was used to assess the quality of life (QOL) of the sample. This measure contains 26 items, which are tabulated to provide four subscales. For the present study only the physical and psychological WHOQOL-BREF were included. The physical QOL subscale consists of seven questions, which are responded to on a 5-point scale from one (very dissatisfied/not at all) to five (very satisfied; an extreme amount/completely/extremely) to determine the physical QOL of the individual over the last 2 weeks. The internal consistency of this scale for the present samples is 0.82 and 0.81 for the diabetes and amputation samples respectively. The psychological QOL subscale consists of six questions, which are responded to on a 5-point scale from 1 (very dissatisfied; never; not at all) to 5 (very satisfied; always; an extreme amount/completely/extremely) to determine the psychological QOL of the individual over the last 2 weeks. Good internal consistency for this scale was found, with Cronbach’s alpha = 0.82 in the diabetes sample and 0.84 for the amputee sample.

#### Body Image Disturbance Questionnaire (BIDQ)

The BIDQ (Cash et al., 2004) was used to assess subjective body image disturbance as defined by body image dissatisfaction, body image distress, and body image dysfunction. The BIDQ contains seven rating scale items that investigate concerns related to appearance, fixation on these concerns, distress associated with these concerns, and impairment and avoidance resulting from these concerns. The measure has established reliability and validity. On the BIDQ, participants rate on a variety of 5-point scales from 1 (not at all concerned/never) to 5 (extremely/very often) the degree to which each of the seven items describes their thoughts or feelings regarding their body. Scores range from 1 to 5, with higher scores reflecting greater body image disturbance. The scale had good internal consistency (Cronbach’s alpha = 0.90 and 0.88 for the diabetes and amputee samples respectively).

#### Appearance Schemas Inventory-Revised (ASI-R)

The ASI-R (Feragen and Stock, 2018) is a 20-item measure, which uses a 5-point scale from 1 (disagree) to 5 (agree) to measure an individual’s psychological investment in her/his physical appearance. The ASI-R has two subscales, Self-Evaluative Salience (SES) and Motivational Salience (MS) and as a result three scores can be obtained; two subscale scores and a composite score. The SES subscale measures the degree of an individual’s investment in their appearance. The MS subscale measures investment in terms of compensatory behaviors (self-management or enhancement of one’s appearance). For the present study only the SES subscale was used, as this was the component of investment that is referred to in White’s model. This scale had good internal consistency (Cronbach’s alpha = 0.73 and 0.65 respectively in this study for the diabetes and amputee groups respectively).

#### Body Image Ideals Questionnaire (BIQ)

The BIQ (Cash and Szymanski, 1995) was included to assess the importance an individual places on the degree to which actual and ideal appearances match. The BIQ is an 11-item scale with two required ratings for each body part. The first asks the respondent to rate on a 3-point scale from 0 (exactly as I am) to 3 (very unlike me) how alike their actual body part/s are to their ideal. The second part of each item asks the respondent to rate the importance of the body part on a 3-point scale from 0 (not important) to 3 (very important). The total score on this measure is calculated as the product of the discrepancy and importance ratings, with higher scores indicative of a greater self-ideal discrepancy. The internal consistency of this measure has been established in the present study, with Cronbach alpha = 0.92 and 0.92 for the diabetes and amputee samples respectively.

### Data Analysis

Pearson bivariate correlations were employed to identify significant demographic, lifestyle and disease correlates of body image dissatisfaction, depressive symptoms, anxiety symptoms, and quality of life. Structural equation modeling was then used to test White’s model of body image. A single latent variable incorporating depression, anxiety, psychological quality of life, and physical quality of life was constructed for the structural model to predict the shared variance in psychosocial outcomes. This one-factor measurement model was tested separately for both the diabetes and amputation groups. The results of these measurement models can be found in Supplementary Material (under subheading 1, Supplementary Data).

For the diabetes group, age, gender, BMI, total medical conditions, and total number of disease related complications were included as covariates in the model, as they were related to at least one of the primary outcome variables (*p* < 0.05). Time since diagnosis and type of DM were not included in the analyses as they were not significantly associated with any of the primary study variables (*p* > 0.05; see Table A of the Supplementary Material). For the amputation group, age, gender, pain, time since amputation, and number of medical conditions were included as covariates as they were related to the primary outcome variables (*p* < 0.05). Prosthesis use was not included as it was not significantly associated with any of the primary study variables (*p* > 0.05; complete data for these bivariate associations are available in Table B of the Supplementary Material).

All model estimations were conducted with AMOS (Version 20), using maximum-likelihood estimation. Model fit was assessed with the chi-square statistic, the root-mean-square error of approximation (RMSEA), comparative fit index (CFI), and Tucker Lewis Index (TLI). These indices assess how much the model-estimated covariance differs from the observed covariance matrix. Acceptable fit is indicated by a RMSEA of 0.08 or less, and CFI and TLI values above 0.9. The use of bootstrapping is recommended for testing mediation, as it does not impose assumptions regarding the normality of the sampling (Preacher and Hayes, 2004). As such, 95% bias corrected confidence intervals were calculated using bootstrapping with 2000 samples.

**FIGURE 1:**
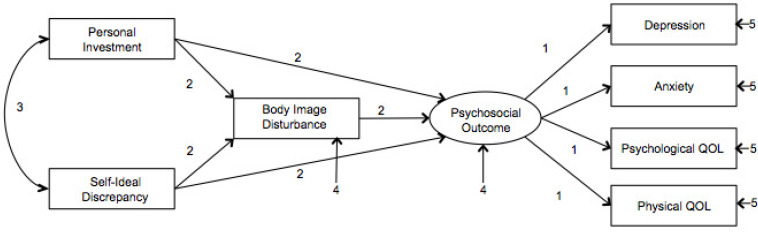
Invariance modeling pathways cumulatively constrained across the model. (1) Measurement Weights are constrained. (2) In addition Structural Weights are constrained. (3) In addition Structural Covariance is constrained. (4) The residual error from body image disturbance and psychosocial outcome are constrained. (5) Residual error from Depression, Anxiety, Psychological QOL, and Physical QOL are constrained.

Model invariance was tested by constraining the model for both groups in five cumulative steps, as illustrated in Figure 1: (1) Measurement Weights, (2) Structural Weights, (3) Structural Covariance, (4) Structural Residuals, and (5) Measurement Residuals. As the covariates differed across the two groups and were not of primary interest in the model they were excluded from the tests of invariance. Model invariance will be evaluated in line with current literature, which indicates that approximate fit indexes such as CFI difference scores should be used when there are large samples being examined. According to Cheung and Rensvold (2002), invariance is indicated using the CFI difference test, where the difference in CFI is less than or equal to 0.01. This approach was chosen as recent reports have indicated that it is a better test of invariance than the chi-square difference test as it is not influenced by sample size (Cheung and Rensvold, 2002; Kline, 2010).

## Results

### Descriptive Statistics

Table 1 outlines the descriptive statistics of participants in the diabetes sample. Individuals with diabetes had a mean age of 64.4 years (*SD* = 12.35; ranging from 21 to 89 years); the majority were male (60%), with Type 2 diabetes (78%), and an average BMI in the overweight range (*M* = 29.21; *SD* = 7.50). Time since diagnosis ranged from 0.1 to 66.0 years (*M* = 14.00; *SD* = 11.90). More than half the sample reported having at least one comorbid medical condition (55.7%), and 52.8% reported having at least one diabetes related complication.

**TABLE 1:** Mean scores and standard deviations for the study measures in individuals with diabetes (*N* = 212).

Measure	Mean	*SD*	Range
HADS-depression	4.16	3.56	0 to 20
HADS-anxiety	5.38	4.17	0 to 18
WHOQOL-psychological	70.61	17.08	13 to 100
WHOQOL-physical	70.06	18.14	11 to 100
BIDQ	1.62	0.74	1 to 4.43
ASI-R (self evaluative)	2.53	0.65	1 to 4.58
BIQ	1.38	1.49	-2.09 to 6.82

Individuals with an amputation (*N* = 227) ranged in age from 20 to 91 years (*M* = 58.54, *SD* = 14.45); the majority were male (70%) and over half of the sample reported at least one comorbid medical condition (64%). Time since amputation ranged from 0.1 to 66.0 years (*M* = 14.92; *SD* = 15.31). Table 2 provides both additional demographic characteristics for the sample, and descriptive statistics for all primary study variables.

**TABLE 2:** Amputation characteristics and mean scores and standard deviations for the measures (*N* = 227).

Characteristic	Frequency		
**Cause of amputation**			
-Trauma	37.4%		
-Diabetes	23.8%		
-Non-diabetes vascular	13.7%		
-Cancer	11%		
-Other (i.e., infection, scleroderma)	14.1%		
**Site of amputation**			
-Unilateral below knee	69%		
-Bilateral below knee	5%		
-Unilateral above knee	26%		
**Prosthesis**			
% with a prosthesis (*N* = 227)	85%		
% daily use (*N* = 193 have prosthesis)	74%		

**Measures**	**Mean**	***SD***	**Range**

Pain (0–10 VAS)	3.82	2.60	0 to 10
HADS-depression	4.72	3.62	0 to 19
HADS-anxiety	5.31	4.00	0 to 20
WHOQOL-psychological	67.96	20.38	0 to 100
WHOQOL-physical	63.36	18.82	0 to 100
BIDQ	2.24	0.88	1 to 5
ASI-R (self evaluative salience)	2.66	0.73	1 to 5
BIQ	1.29	1.58	-1.82 to 6.55

*VAS, Visual Analog Scale; HADS, Hospital Anxiety and Depression Scale; WHOQOL, World Health Organization Quality of Life; BIDQ, Body Image Disturbance Questionnaire; ASI-R (SES), Appearance Schemas Inventory-Revised (Self Evaluative Salience subscale); BIQ, Body Image Ideals Questionnaire.*

### Correlations

In both groups, all correlations were statistically significant. Bivariate associations between the primary study variables are available in Tables C and D of the Supplementary Material, for the diabetes and amputation groups, respectively.

### Test of the Structural Models

The structural model was constructed to examine the overall fit of the model and to test for body image disturbance as a mediator of both personal investment and self-ideal discrepancy, with psychosocial outcome. The structural model for individuals with diabetes, included age, gender, body mass index (BMI), number of diabetes related complications, and medical conditions as covariates. This model accounted for 45% of the variance in psychosocial outcome and the results indicated good model fit (χ^2^(25) = 48.80, *p* = 0.003; CFI = 0.974, TLI = 0.917, and RMSEA = 0.067). For the amputation sample, age, gender, time since amputation, pain, and medical conditions were included as covariates in the model. This model accounted for 64% of the variance in psychosocial outcome and the results indicated good fit (χ^2^(23) = 54.73, *p* = 0.000; CFI = 0.967, TLI = 0.906, and RMSEA = 0.078).

**FIGURE 2:**
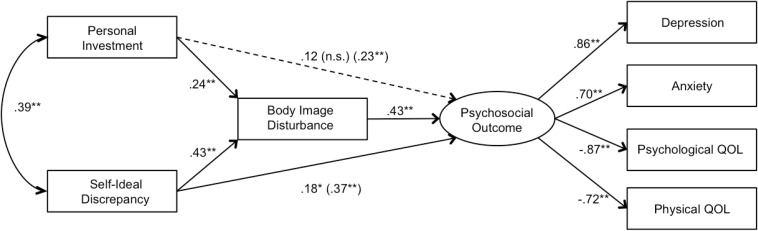
Standardized path coefficients of the structural model for individuals with diabetes, evaluating the mediating role of body image disturbance for self-ideal discrepancy and personal investment on psychosocial outcome for individuals with diabetes (*N* (212). Age, gender, BMI, number of medical conditions, and diabetes related complications were included as covariates of all variables. Unmediated path coefficients for personal investment and self-ideal discrepancy on psychosocial outcome are presented in parentheses for comparison with the mediated path coefficients. The dotted line indicates full mediation in the mediational model. ^∗^*p* (0.05), ^∗∗^*p* (0.01).

**FIGURE 3:**
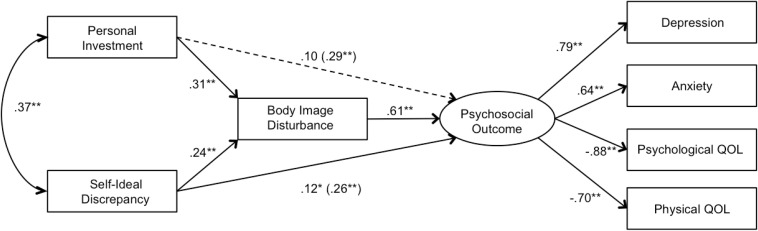
Standardized path coefficients of the structural model evaluating the mediating role of body image disturbance for self-ideal discrepancy and personal investment on psychosocial outcome for individuals with an amputation (*N* (227). Age, gender, pain, time since amputation, and number of medical conditions were included as covariates of all variables. Unmediated path coefficients for personal investment and self-ideal discrepancy on psychosocial outcome are presented in parentheses for comparison with the mediated path coefficients. The dotted line indicates full mediation in the mediational model. ^∗^*p* (0.05), ^∗∗^*p* (0.01).

To test the mediating role of body image disturbance, bootstrap estimates were conducted. For individuals with diabetes, results indicated full mediation of the relationship between personal investment and psychosocial outcome via body image disturbance (estimate = 0.102; 95% bias corrected CI [0.04;0.19]), and partial mediation of the relationship between self-ideal discrepancy and psychosocial outcome via body image disturbance (estimate = 0.185, 95% CI [0.10;0.29]). See Figure 2 for the unmediated and mediated effects. Similarly, for individuals with an amputation, bootstrap estimates revealed significant results for both the mediation effects in the model; with results indicating full mediation of the relationship between personal investment and psychosocial outcome via body image disturbance (estimate = 0.189; 95% bias corrected CI [0.12;0.27]), and partial mediation of the relationship between self-ideal discrepancy and psychosocial outcome via body image disturbance (estimate = 0.150, 95% CI [0.07;0.23]). See Figure 3 for the unmediated and mediated effects.

### Comparison of the Variance Between Groups

We compared the total model variance accounted for in both body image disturbance and psychosocial outcome in the diabetes and amputation groups using the Fisher exact *z*-test. For subjective body image disturbance, total variance accounted for did not significantly differ for the model of individuals with diabetes (*R*^2^ = 0.517) versus individuals with an amputation (*R*^2^ = 0.458; *z* = 0.853, *p* = 0.39). For psychosocial outcomes, total variance accounted for was significantly greater for the model of individuals with an amputation (*R*^2^ = 0.640) compared to individuals with diabetes (*R*^2^ = 0.446; *z* = 3.031, *p* = 0.002).

### Invariance Models

When compared to the unconstrained models (see Table 3), stepwise placing constraints at the measurement weights, structural weights (the causal pathways to body image disturbance and psychosocial outcome), structural covariance (between personal investment and self-ideal discrepancy), and measurement residuals resulted in no loss of model fit according to any of the fit statistics (ΔCFI < 0.01). This supports structural invariance across the diabetes and amputation samples. When compared to the unconstrained models, adding constraints for the structural residuals (error terms for psychosocial outcome and body image disturbance) and measurement residuals (error terms for the indicator variables predicting the psychosocial outcomes factor) resulted in lower model fit (ΔCFI > 0.01). These results indicate that the model was equivalent across both groups with the exception of the error terms. To rule out the role of shared illness (diabetes), invariance modeling was re-analyzed using only those individuals with an amputation without diabetes (*N* = 173) and the group with diabetes without amputation (*N* = 212). Results from this additional analysis provided an identical pattern of results, including invariance between the models, which confirms that any shared characteristics of the original groups cannot account for the invariance of the model.

**TABLE 3:** Summary of testing of invariance of the structural model across individuals with diabetes (*N* = 212) and individuals with an amputation (*N* = 227).

Invariance model	*(*^2^	df	RMSEA	CFI	TLI	AIC
Unconstrained model	75.12	22	0.074	0.965	0.933	143.13
1. Measurement weights	85.46	25	0.074	0.960	0.933	147.46
2. Structural weights	94.34	30	0.070	0.958	0.941	146.34
3. Structural covariance	97.43	33	0.067	0.958	0.946	143.43
4. Structural residuals	97.43	35	0.073	0.946*	0.935	158.89
5. Measurement residual	127.45	39	0.072	0.942	0.937	161.45

RMSEA, root mean squared error of approximation; CFI, comparative fit index; TLI, Tucker-Lewis index. *ΔCFI > 0.01.

## Discussion

Overall, the aim of the study was to test a well-known theoretical model of body image disturbance in health, in two distinct samples. To this end, we chose individuals with diabetes and individuals with an amputation, who differ importantly on the degree of objective and visible bodily change. Results from both groups indicate support for White’s (2000) model for use outside of oncology. Specifically, as predicted, body image disturbance, personal investment, and self-ideal discrepancy all independently and directly predicted psychosocial outcome, over and above demographic and medical factors. Consistent with our hypotheses the structural model explained greater variance in psychosocial outcome for individuals with amputation than for individuals with diabetes. These findings are consistent with the findings of previous research. Notably, it appears that the visible difference associated with a condition (in this case amputation) influences the degree of body image disturbance experienced. There was evidence to support the hypothesis that the relationship between personal investment and psychosocial outcomes was mediated by body image experience for both groups, and there was also evidence that body image experience partially mediated the relationship between importance weighted self-ideal discrepancy and psychosocial outcome in both groups.

Finally, the study also examined the hypothesis that the model would be equivalent across the two groups. It was found that despite the distinct difference between the two groups, there was structural invariance across the two groups. That is, the theoretical model of direct and indirect relationships among variables was the same. Only the structural residuals varied across the two groups, meaning that the explanatory power of the model in predicting body image disturbance and psychosocial outcome differed between groups, as predicted. Importantly, these results were also found when the analyses were run with purely independent models (i.e., anyone with an amputation removed from the diabetes group and anyone with diabetes removed from the amputation group). The structural invariance highlights that the pattern of the core relationships between subjective body image disturbance, personal investment, self-ideal discrepancy, and psychosocial outcome are the same among groups of individuals with a chronic illness (diabetes) and individuals with a disability (amputation).

Before the implications of these findings can be considered it is important to bear in mind the limitations of the present study. First, as the design was cross-sectional, causal inferences cannot be made from these results. To test the causal inferences, prospective designs are needed. Secondly, the two samples are samples of convenience. It is likely that they differ in important ways from the population of people who have an amputation. For example, the rate of people in the amputation group, whose amputation was due to diabetes is lower than would be expected. On the one hand, this will limit the generalizability of the samples. However, on the other hand, there is no reason to think that the pattern of relationships between variables would be affected and these relationships were the primary interest in the present study. Furthermore, had a very high proportion of the amputation sample had diabetes, then these samples would not have had sufficient independence to answer the research questions. Third, self-reported BMI in the diabetes sample was used; therefore it is possible that this is not entirely accurate. Importantly, self-reported BMI is highly correlated with actual weight (Stunkard and Albaum, 1981). Further, it was not possible to control for BMI in the amputee group because its validity and meaning would vary depending on the nature and site of amputation. Finally, because we examined two different samples and investigated invariance of the models, we were unable to include illness-specific constructs, such as diabetes-related distress or fear of hypoglycemia. Therefore, we cannot comment on the role of body image in these important constructs.

These limitations notwithstanding, there are a number of important strengths of the present study. The present study was constructed to examine the validity of White’s (2000) model for other medical groups. Two large samples were recruited, which enabled both the inclusion of covariates and the use of structural equation modeling to examine the model in a systematic manner. In contrast to much of the previous research, the present study also utilized well-validated measures of both body image variables and psychosocial outcome. The use of large cohorts, sophisticated analyses, and controlling for covariates enable us to be confident about the findings of this study.

The present study provides preliminary empirical support that White’s (2000) cancer-specific model of body image applies to other medical groups. White’s model has been influential in the study of body image in people living beyond cancer and has been applied in a few other illnesses (e.g., multiple sclerosis; Wilski et al., 2016) but has not been applied to those following amputation or people with diabetes. It was found that body image experience, self-ideal discrepancy and personal investment all predict psychosocial outcome, though the amount of variance accounted for by the model is larger in the group with an amputation when compared to the diabetes group. This provides further evidence to support the contention that there is a direct relationship between objective change in appearance and body image disturbance, such that body image variables are more strongly associated with psychosocial outcome in groups where objective changes are caused by the illness or its treatment. However, the hypothesized relationships were nonetheless consistent across the two samples. That is, body image experience significantly mediated the relationship between personal investment and psychosocial outcome and partially mediated the relationship between self-ideal discrepancy and psychosocial outcome. The consistency of these relationships was also confirmed through our tests of invariance. These results underscore the importance of the body image experience of an individual irrespective of the degree of objective physical changes associated with their illness, disability, change in appearance or related treatment.

These results have important clinical implications. The strong relationship between body image and psychosocial outcomes suggests that body image could be an important target for intervention for individuals with diabetes and individuals with an amputation. Body image accounted for large proportions of the variance in the latent variable of psychosocial outcome, which consisted of depression, anxiety, physical, and psychological quality of life. Clearly, future research needs to test this model in other health groups to determine the generalizability of the model across illnesses. In addition, future research should utilize prospective designs to determine whether body image holds a causal relationship with psychosocial outcome for health groups. Available research, however, confirms that body image does predict future depression (Moreira and Canavarro, 2010; Bullen et al., 2012), anxiety (Sharpe et al., 2011; Bullen et al., 2012), psychological distress (Carver et al., 1998; Sharpe et al., 2011), and poorer quality of life (Shimozuma et al., 1999; Fauerbach et al., 2000; Browall et al., 2008; Taylor-Ford et al., 2012) in patients with physical health conditions. However, if confirmed, these results suggest that interventions that target body image may be of particular relevance not only for people following amputation, but also for people with diabetes. Cognitive behavioral interventions for body image disturbance have shown promise (Cash, 2002) and thus a clinical direction for future research would be to examine the efficacy of such therapeutic approaches for body image in health groups. Notwithstanding the need for future research, it is clear from the findings of the present study that body image is an important factor for individuals with diabetes and individuals with an amputation and thus it is important that body image becomes a focus of future health research and clinical interventions, rather than being a relatively neglected topic.

## Data Availability Statement

The raw data supporting the conclusions of this article will be made available by the authors, without undue reservation.

## Ethics Statement

This study was approved by The University of Sydney’s Human Research Ethics Committe and the local health district Human Research Ethics Committee. The patients/participants provided their written informed consent to participate in this study.

## Author Contributions

SM developed the research question, collected the data, analyzed the data, interpreted findings, and worked on the manuscript. LS developed the research question, analyzed the data, interpreted findings, and worked on the manuscript. CM assisted with analyzing the data and worked on the manuscript. AB assisted with research question, interpreting findings, and worked on the manuscript. All authors contributed to the article and approved the submitted version.

## Conflict of Interest

The authors declare that the research was conducted in the absence of any commercial or financial relationships that could be construed as a potential conflict of interest.
