# Supplementary material for: The Role of Body Image on Psychosocial Outcomes in People With Diabetes and People With an Amputation
Source: Front Psychol. 2021 Jan 11;11:614369. doi: 10.3389/fpsyg.2020.614369 (PMC7829667; doi:10.3389/fpsyg.2020.614369)
Supplement: Supplementary file 1 [file Data_Sheet_1.docx]

Supplementary Material

# Supplementary Data

*Results of the Measurement Model for Psychosocial Outcome for Individuals with Diabetes*

The measurement model was constructed to form a single psychosocial outcome variable, which incorporated depression, anxiety, psychological quality of life and physical quality of life. Standardized estimates of the factor loadings for depression (*β=* .91), anxiety (*β=* .66), psychological quality of life (*β=* -.86) and physical quality of life (*β=* -.74) on psychosocial outcome were all significant at *p* < .001. Fit indices were acceptable for CFI and TLI, but somewhat high for RMSEA: *χ^2^*(2)= 11.53, *p* = .003; CFI = .979, TLI= .936, and RMSEA= .150. Modification indices suggested that the correlated error between anxiety and physical QOL was a source of misfit in the model, however when re-modeled covarying these factors, RMSEA remained somewhat high: *χ^2^*(1)= 4.15, p= .042; CFI= .993, TLI= .958, and RMSEA= .122 and thus the first model was used in the structural model.

*Results of the Measurement Model for Psychosocial Outcome for Individuals with an Amputation*

Standardized estimates of the factor loadings for depression (*β=* .82), anxiety (*β=* .68), psychological quality of life (*β=* -.87) and physical quality of life (*β=* -.78) were all significant at *p* < .001. Fit indices were acceptable fit for CFI and TLI, but somewhat high for RMSEA: *χ^2^*(2)= 10.27, p= .006; CFI= .981, TLI= .944, and RMSEA= .135. As in the diabetes sample, modification indices suggested that the correlated error between anxiety and physical QOL was the source of the misfit. However when correlated error was included in the model, the measurement model overfit the data: *χ^2^*(1)= .079, p= .779; CFI= 1.00, TLI= 1.01, and RMSEA= .000. As such the first model was utilised in the structural model.

# Supplementary Tables

Table A.

*Bivariate Associations Between the Primary Study Variables and Demographic and Medical Variables for Individuals in the Diabetes Group*

| Variable | Depression | Anxiety | Psych. QOL | Physical QOL | Body Image Disturbance | Personal Investment | Self-ideal Discrepancy |
| --- | --- | --- | --- | --- | --- | --- | --- |
| Age | -.139* | -.298** | -0.048 | .238** | -.153* | -.135* | -.204** |
| Gender (1= M; 2= F) | 0.02 | 0.117 | -0.109 | -0.096 | .138* | .141* | .195** |
| Body Mass Index | .270** | .213** | -.298** | -.231** | .397** | .234** | .362** |
| Time since diagnosis | 0.007 | -0.065 | 0.048 | 0.068 | -0.087 | -0.029 | -0.119 |
| Diagnosis Type  (1= Type 1; 2= Type 2) | 0.028 | -0.022 | -0.101 | 0.009 | 0.096 | -0.022 | 0.045 |
| Number of Diabetes- Related Complications | .180** | -0.008 | -.221** | -0.063 | .183** | 0.015 | 0.126 |
| Number of Additional Medical Conditions | 0.09 | 0.04 | -.212** | -0.074 | 0.077 | -0.107 | .146* |

*Note.* QOL= Quality of Life. **p < .05.* ***p < .01*

Table B

*Bivariate Associations Between the Primary Study Variables and Demographic and Medical Variables for Individuals in the Amputation Group*

| Variable | Depression | Anxiety | Psych. QOL | Physical QOL | Body Image Disturbance | Personal Investment | Self-ideal Discrepancy |
| --- | --- | --- | --- | --- | --- | --- | --- |
| Age | 0.029 | -.224** | -.165* | .133* | -0.1 | -.224** | -0.124 |
| Gender (1= M; 2= F) | -0.051 | 0.061 | 0.041 | -0.019 | 0.105 | .196** | 0.105 |
| Number of Additional Medical Conditions | .270** | .213** | -.298** | -.231** | .397** | .234** | .362** |
| Pain | .314** | .357** | -.471** | -.338** | .467** | .204** | .216** |
| Time Since Amputation | -.200** | -.194** | .216** | .148* | -0.104 | -0.048 | -0.074 |
| Prosthetic Use  (0=Not everyday, 1= Everyday) | -0.038 | -0.035 | 0.079 | 0.022 | -0.029 | -0.024 | -0.062 |

*Note.* QOL= Quality of Life. **p < .05.* ***p < .01*

Table C.

*Bivariate Associations Among the Primary Study Variables in Study 1 (N=212)*

| Variable | 1 | 2 | 3 | 4 | 5 | 6 | 7 |
| --- | --- | --- | --- | --- | --- | --- | --- |
| 1. Depression | ----- |  |  |  |  |  |  |
| 2. Anxiety | .592** | ----- |  |  |  |  |  |
| 3. Psychological QOL | -.769** | -.623** | ----- |  |  |  |  |
| 4.Physical QOL | -.698** | -.413** | .624** | ----- |  |  |  |
| 5. Body Image Disturbance | .540** | .472** | -.517** | -.571** | ----- |  |  |
| 6. Personal Investment | .303** | .395** | -.378** | -.276** | .465** | ----- |  |
| 7. Self-Ideal Discrepancy | .460** | .421** | -.478** | -.443** | .651** | .425** | ----- |

*Note.* QOL= Quality of Life. **p < .05.* ***p < .01*

Table D.

*Bivariate Associations Among the Primary Study Variables in Study 2 (N=227)*

| Variable | 1 | 2 | 3 | 4 | 5 | 6 | 7 |
| --- | --- | --- | --- | --- | --- | --- | --- |
| 1. Depression | ----- |  |  |  |  |  |  |
| 2. Anxiety | .584** | ----- |  |  |  |  |  |
| 3. Psychological QOL | -.694** | -.619** | ----- |  |  |  |  |
| 4.Physical QOL | -.665** | -.464** | .689** | ----- |  |  |  |
| 5. Body Image Disturbance | .589** | .572** | -.669** | -.641** | ----- |  |  |
| 6. Personal Investment | .246** | .389** | -.460** | -.350** | .501** | ----- |  |
| 7. Self-Ideal Discrepancy | .324** | .362** | -.454** | -.398** | .479** | .438** | ----- |

*Note.* QOL= Quality of Life. **p < .05.* ***p < .001*
